# Self-Regulation of SMR Power Led to an Enhancement of Functional Connectivity of Somatomotor Cortices in Fibromyalgia Patients

**DOI:** 10.3389/fnins.2020.00236

**Published:** 2020-03-19

**Authors:** Juan L. Terrasa, Alfonso Barros-Loscertales, Pedro Montoya, Miguel A. Muñoz

**Affiliations:** ^1^Cognitive and Affective Neuroscience and Clinical Psychology, Research Institute of Health Sciences (IUNICS) and Balearic Islands Health Research Institute (IdISBa), University of the Balearic Islands (UIB), Palma, Spain; ^2^Department of Psychology, Universitat Jaume I, Castellón, Spain; ^3^Brain, Mind and Behavior Research Center, University of Granada (CIMCYC-UGR), Granada, Spain

**Keywords:** fibromyalgia, neurofeedback, sensorimotor rhythm, fMRI, functional connectivity, somatosensory cortex, motor cortex

## Abstract

Neuroimaging studies have demonstrated that altered activity in somatosensory and motor cortices play a key role in pain chronification. Neurofeedback training of sensorimotor rhythm (SMR) is a tool which allow individuals to self-modulate their brain activity and to produce significant changes over somatomotor brain areas. Several studies have further shown that neurofeedback training may reduce pain and other pain-related symptoms in chronic pain patients. The goal of the present study was to analyze changes in SMR power and brain functional connectivity of the somatosensory and motor cortices elicited by neurofeedback task designed to both synchronize and desynchronize the SMR power over motor and somatosensory areas in fibromyalgia patients. Seventeen patients were randomly assigned to the SMR training (*n* = 9) or to a sham protocol (*n* = 8). All participants were trained during 6 sessions, and fMRI and EEG power elicited by synchronization and desynchronization trials were analyzed. In the SMR training group, four patients achieved the objective of SMR modulation in more than 70% of the trials from the second training session (good responders), while five patients performed the task at the chance level (bad responders). Good responders to the neurofeedback training significantly reduced pain and increased both SMR power modulation and functional connectivity of motor and somatosensory related areas during the last neurofeedback training session, whereas no changes in brain activity or pain were observed in bad responders or participants in the sham group. In addition, we observed that good responders were characterized by reduced impact of fibromyalgia and pain symptoms, as well as by increased levels of health-related quality of life during the pre-training sessions. In summary, the present study revealed that neurofeedback training of SMR elicited significant brain changes in somatomotor areas leading to a significant reduction of pain in fibromyalgia patients. In this sense, our research provide evidence that neurofeedback training is a promising tool for a better understanding of brain mechanisms involved in pain chronification.

## Introduction

Fibromyalgia (FM) is a chronic pain syndrome characterized by generalized and enhanced pain sensitivity, as well as by fatigue, morning stiffness, sleep disturbance, affective and cognitive dysfunctions, and a generalized hypersensitivity to pain stimulation (Wolfe et al., 1990, 1995). Its prevalence ranges from 3 to 10% in the general adult population and is more frequent in women than men (Wolfe et al., 2013; Clauw et al., 2018). Although the underlying etiology of FM still remains unclear, several studies have showed altered brain activation in areas of the so called pain network involved in the emotional and cognitive processing of pain (Gracely, 2004; Burgmer et al., 2009, 2010). Functional magnetic resonance imaging (fMRI) studies have further reported that resting-state functional connectivity of the anterior cingulate cortex (ACC), basal ganglia insula, thalamus, amygdala, medial prefrontal cortex and somatosensory and motor cortices were increased in patients with FM as compared with healthy controls (Cifre et al., 2012; Flodin et al., 2014; Ichesco et al., 2014), reflecting an abnormal hyperexcitability of the central nervous system (Desmeules et al., 2003). In this regard, altered somatosensory and motor cortex activity has been proposed to play a central role in the experience of pain and its chronification (Harris, 1999; Pujol et al., 2014; González-Roldán et al., 2016; Lim et al., 2016; Don et al., 2019). Furthermore, primary motor cortex activity has been suggested to be a major modulator of pain processing (Castillo Saavedra et al., 2014), while primary somatosensory cortex is highly involved in the localization and discrimination of pain experience (Diers, 2019).

EEG neurofeedback is a technique based on learning to self-regulate several parameters of cortical activity such as amplitude, frequency and/or coherence of EEG signal (Gruzelier, 2014). During neurofeedback training, individuals learn to modify their own brain activity by receiving visual or acoustic information about these EEG parameters (Enriquez-Geppert et al., 2017). Neurofeedback has been widely used successfully in chronic pain syndromes, showing potential benefits to reduce pain, anxiety and depression in these patients (Mueller et al., 2001; Kravitz et al., 2006; Kayıran et al., 2010; Nelson et al., 2010; Caro and Winter, 2011). In this sense, it has been highlighted that the efficacy of the treatment could be related to the decrease and/or the increase of somatosensory and motor activity associated with the processing of nociceptive information (Jensen et al., 2008, 2014). Sensorimotor rhythm (SMR) refers to oscillations between 12 and 15 Hz recorded over somatosensory and motor areas (Pfurtscheller and Lopes da Silva, 1999; Budzynski, 2009; Gruzelier, 2014). Studies about SMR-based neurofeedback training in chronic pain patients have shown significant short-term improvements in pain relief, and other non-pain associated symptoms in patients with complex regional pain syndrome (Jensen et al., 2007), chronic low back pain (Mayaud et al., 2019) and chronic spinal cord injury (Vučković et al., 2019). Furthermore, SMR-based neurofeedback training has provided evidence that it is able to reduce pain and fatigue symptoms in patients with fibromyalgia (Kayıran et al., 2010; Caro and Winter, 2011).

Although several studies have demonstrated that neurofeedback training was able to reduce pain-related symptoms, little is known about the functional changes that the SMR-based neurofeedback training is eliciting in EEG activity and brain connectivity. In this sense, increased resting-state functional connectivity in several pain areas such as ACC (Ros et al., 2013), insula (Kluetsch et al., 2014) or the amygdala (Nicholson et al., 2016) have been reported after neurofeedback training of the alpha EEG. In addition, a significant enhancement of resting-state functional connectivity of somatosensory and motor cortices has been demonstrated after neurofeedback training of SMR in patients with stroke (Várkuti et al., 2013; Young et al., 2014; Mohanty et al., 2018) and in healthy participants (Terrasa et al., 2019). Although all these findings support the notion that neurofeedback training can produce relevant changes in clinical symptoms and brain activity, little is known about the neurophysiological processes involved during brain self-regulation training.

The primary goal of the present study was to analyze changes in SMR activity and brain functional connectivity of the somatosensory and motor cortices in response to neurofeedback training of the SMR in FM patients. For this purpose, a training protocol based on learning to synchronize and desynchronize the SMR power over motor and somatosensory areas was applied during six sessions, and brain changes produced when performing the neurofeedback task were examined. Our hypothesis was that those FM participants achieving a successful self-regulation of the SMR would show increased synchronization and desynchronization modulation of SMR power, enhanced somatomotor functional connectivity and reduced pain during the last neurofeedback training session.

## Materials and Methods

### Participants

Seventeen right-handed female patients (aged 54.94 ± 10.11) with a diagnosis of FM were recruited from the *Asociación Granadina de Fibromialgia* (AGRAFIM) in Granada (Spain). The diagnosis of FM was confirmed by a professional rheumatologist following the American Rheumatology College 2010 Criteria. Exclusion criteria were: FM diagnosis of less than 1 year, pregnancy, vision or auditory deficits, and neurological or psychiatric diseases (except depression). Thirteen of the seventeen fibromyalgia patients had a diagnosed and medicated depression disorder. No participants with other psychiatric disorders were accepted in the study. All participants were taking regular medication, including analgesic/myorelaxant (88.24%), antidepressant (76.47%), and anxiolytic (70.59%). During the experiment, participants were asked to avoid the use of any other non-pharmacology therapy. The study was conducted in accordance with the Declaration of Helsinki (1991) and approved by the Ethics Committee of the Balearic Islands (Spain). Written informed consents were obtained from the participants after the experimental procedure explanation.

### Procedure and Clinical Assessment

The patients attended a total of seven sessions and were sequentially assigned to either a SMR neurofeedback training (SMR, *n* = 9) or a control group that received false feedback during the training task (SHAM, *n* = 8) following the order of their arrival at the first session. In the first session, a thorough psychological evaluation was conducted under the supervision of a trained and experienced psychologist (MM), including a semi-structured interview on chronic pain and following self-report questionnaires: the McGill Pain Questionnaire (MPQ) (Melzack, 1975), the West Haven-Yale Multidimensional Pain Inventory (WHYMPI) (Kerns et al., 1985), the Tampa Scale for Kinesiophobia (TSK) (Roelofs et al., 2004), the Pain Anxiety Symptoms Scale (PASS) (McCracken et al., 1992), the Pain Vigilance and Awareness Questionnaire (PVAQ) (McCracken, 1997), the MOS Social Support Survey (MOS) (Sherbourne and Stewart, 1991), the Coping Strategies Questionnaire (CSQ) (Rosenstiel and Keefe, 1983), the MOS 36-item Short-form Health Survey (SF-36) (Ware and Sherbourne, 1992), the Beck Depression Inventory (BDI-II) (Beck et al., 1996), and the Fibromyalgia Impact Questionnaire (FIQ) (Burckhardt et al., 1991).

After the psychological assessment, patients participated in a six-session neurofeedback training program with 3 sessions per week during 2 weeks. The training protocol was successfully tested in a previous work with healthy participants (Terrasa et al., 2019). During the first (PRE session) and the sixth sessions (POST session), all individuals performed the training in a MRI scanner, while the rest four sessions were performed in a MRI simulator. The simulator reproduced the characteristic disturbing sounds of the real scanner. At the end of the PRE and the POST sessions, patients were asked to rate their pain using a numerical scale ranging from 0 to 100. Furthermore, given that high anxiety levels can impair neurofeedback training (Hardman et al., 1997; Gruzelier et al., 1999), the level of anxiety was assessed with the State-Trait Anxiety Inventory (STAI) (Spielberger et al., 1970) before the beginning of each assessment session. In addition, all participants were asked to complete a diary three times a day (morning, afternoon, evening) with ratings of pain, fatigue and negative mood on a numerical scale ranging from 0 to 100.

### EEG Neurofeedback Task and Processing

During the neurofeedback training program, EEG signals were acquired by a QuickAmp amplifier (Brain Products GmbH, Munich, Germany) at 1000 Hz sampling rate, with high-pass and low-pass filter settings at 0.10 and 70 Hz, respectively. A 50 Hz notch filter was also applied. EEG was recorded from 64 Ag/AgCl electrodes placed according of the 10-20 International System referenced to FCz. Ground electrode was located at position AFz. Electrode impedance was kept lower than 10 kOhm.

The EEG neurofeedback task was to learn to synchronize (i.e., by increasing power amplitudes at specific electrodes) and to desynchronize (i.e., by decreasing power amplitudes at specific electrodes) the SMR. The task was performed using the Cursor Task module of BCI2000 platform (Schalk et al., 2004). Each trial began with the presentation of a target (a gray vertical rectangle) located on the left or right edge of the screen. At the same time, a gray ball appeared in the center of the screen and subjects were asked to control the movement of the ball on the horizontal axis by synchronizing or desynchronizing the SMR for a maximum of 9 s. The goal of the task was to move the ball and impact the target. If the goal was achieved, the ball remained on the screen for a second (reward presentation) and then disappeared; otherwise, the ball simply disappeared. The participants did not receive any instruction other than that they had to learn to control the ball (move it to the right or left according to the position of the target) and hit the target as many times as possible. Two parameters of the task performance were analyzed: number of trials in which the ball hit the target (percentage of hits) and the time to hit the target in successful trials (duration of successful trials).

During each neurofeedback trial, SMR power at C3, CP1, and CP5 electrodes was calculated every 0.5 s of input data by means of maximum entropy method (autoregressive model order = 16) with 3 Hz bin resolution. These signal features were translated into output control signal using a linear equation selecting the power of the selected electrodes into 12–15 Hz frequency bin. Finally, the signal was normalized to make the output control signal zero mean and unit variance. The subjects had to synchronize the SMR power to move the cursor to the left or to desynchronize the power to move the cursor to the right. The greater the power variation was, the greater the cursor movement speed.

Given that the PRE and POST sessions were conducted in the MRI scanner, BrainVision RecView software was applied online to partially remove the gradient artifact (imaging artifact) and the pulse artifact (ballistocardiographic artifact) of MRI from the EEG signal using an automated implementation of the average subtraction method (Allen et al., 1998, 2000). RecView was modified to enable export of the corrected EEG data in real time through a TCP/IP socket to BCI2000. This procedure was optimized with BrainVision Syncbox ensuring an optimal communication between the MRI scanner master clock and Review.

During the PRE and POST sessions, the task consisted of 100 trials (50 trials with the target displayed on each side of the screen) presented in random order with an interval between the 15 s trials, and all participants (SMR and SHAM) received real feedback on their performance. The remaining four training sessions consisted of four runs with 20 trials (10 trials with the target displayed on each side of the screen) presented in random order within each run and with an interval between trials of 6 s. In these neurofeedback training sessions, only the SMR group received real feedback on the SMR power variations, while the SHAM group received random feedback. For the latter, the movement of the ball was manipulated to reach the target only in 50% of the trials (25% right, 25% left).

The preprocessing of EEG data during the PRE and POST sessions was carried out using Matlab R2016b. EEG signals were bandpass filtered within 1–30 Hz and an algorithm for ocular correction (Gratton and Coles) was applied. Data were segmented into epochs of 9 s and separated by trial type (synchronization or desynchronization). Then, power spectral density was calculated for the interval between 1 and 30 Hz (1 Hz resolution) for all channels and each trial type (synchronization or desynchronization). The average power density at C3, CP1, and CP5 electrodes within SMR range (12–15 Hz) was computed and the difference on SMR power between synchronization and desynchronization trials was calculated as an SMR modulation score, reflecting the degree of self-control over SMR activity.

### Functional MRI Data Acquisition

During the PRE and POST sessions, fMRI images were acquired using a 3.0 Tesla scanner (SIEMENS MAGNETOM TrioTim syngo MR). Echo-planar sequence (EPI) functional images of the whole brain (except the cerebellum) were acquired during the EEG neurofeedback for a maximum time of 40 min and 6 s (Total volumes = 1200; 27 axial slices per volume interleaved; TR = 2.0 s; ET = 23 ms; Flip Angle = 80°; Acquisition Matrix = 66 × 66; FOV = 232 mm; Slice Thickness = 3.0 mm; no gap). Furthermore, MPRAGE sequence T1 anatomical images were also acquired for each subject to perform co-register and nuisance pre-analyses (176 slices; TR = 1900 ms; ET = 2.52 ms; Flip Angle = 9°; FOV = 250 mm; Slice Thickness = 1 mm).

### Data Analyses

**FIGURE 1:**
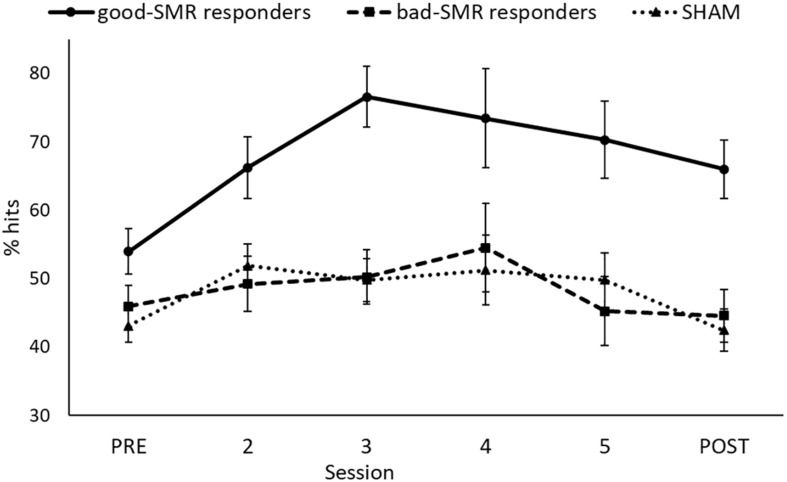
Percentage of successful trials for each group through all the sessions.

After the initial statistical analyses, we observed that participants in the SMR group as a whole could not achieve an average performance above the random level, as demonstrated in previous studies with healthy participants (Cincotti et al., 2008; Blankertz et al., 2010; Terrasa et al., 2019). Furthermore, there were no significant differences between the SMR and the SHAM groups on percentage of hits. Therefore, we decided to subdivide the SMR group in good responders (who achieved a mean performance level above 50% of success during all the sessions) and bad responders (who achieved a mean performance level under 50% of success during all the sessions). Good responders showed 70% of success in at least one session of the neurofeedback training. Thus, the study was finally conducted with three groups: good-SMR responders (*n* = 4) with 67.76% ± 15.97 of successful trials (mean of the six sessions), bad-SMR responders (*n* = 5) with 48.31% ± 7.26 of successful trials and SHAM group (*n* = 8). The task performance (percentage of hits) for each group through the six sessions are shown in Figure 1.

Statistical analyses were carried out using IBM SPSS Statistics v21. For repeated measures analyses, normal distributions of the used variables were tested and Greenhouse–Geisser epsilon corrections were applied to control for violation of the sphericity assumption. Results are reported with the original degrees of freedom, the *p-*values and the partial eta squared parameters (η_p_^2^). When significant effects were found, *post hoc* analyses were performed using Bonferroni correction.

For the demographic and psychological data, one-way analyses of variance (ANOVAs) were used to examine differences among groups (good-SMR responders, bad-SMR responders and SHAM) on age, years since FM was diagnosed and self-report questionnaires (MPQ, WHYMPI, TSK, PASS, PVAQ, MOS, CSQ, SF-36, BDI-II, and FIQ). Group differences in depression comorbidity were analyzed with a Chi-Squared test. Differences on pain ratings and STAI-S scores were examined by using an ANOVA with the factors Group and Assessment session (PRE vs. POST). With respect to the diary data, the average of the three data points (morning, afternoon, night) obtained during the day after the PRE assessment session, as well as the average of the data points obtained the previous day to the POST assessment session was computed for pain, fatigue and negative mood. Differences on these ratings were tested by using ANOVAs with the factors Group and Assessment session (PRE vs. POST).

Task performance scores (percentage of hits and duration of successful trials) during the assessment sessions (PRE vs. POST) were tested by using an ANOVA with the factors Group, Assessment session and Trial type (synchronization vs. desynchronization). Regarding the EEG analyses and to test that good-SMR responders would show significant training effects on SMR modulation scores compared to bad-SMR responders and SHAM group, an ANOVA with the factors Group Assessment Session (PRE vs. POST) was carried out at selected electrodes (C3, CP1, and CP5).

In order to further explore the possible interference of FM symptoms on training effects, those questionnaire scores that showed significant differences among the groups were correlated with task performance scores (percentage of hits and duration of successful trials) and SMR modulation scores during the PRE and POST sessions.

### Functional MRI Analyses

The fMRI connectivity analyses were performed with the CONN-fMRI fc toolbox v18a (Whitfield-Gabrieli and Nieto-Castanon, 2012) in conjunction with SPM 12 (Wellcome Department of Imaging Neuroscience, London, United Kingdom)^1^. All structural and functional sequences in both PRE and POST sessions were pre-processed using the CONN’s default pipeline for volume-based analysis following these steps: resampling to 2 × 2 × 2 mm voxels and unwarping, centering, slice time correction, normalization to the Montreal Neurological Institute (MNI) template, outlier detection to use as a first-level nuisance covariate (ART-based scrubbing), and smoothing to an 8 mm Gaussian kernel. Motion parameters (translations in the x, y, and z directions) were entered as multiple regressors and images with motion over 2.0 mm were regressed entirely out of the time course. Furthermore, BOLD data underwent a denoising process by using CompCor method (Behzadi et al., 2007) in a single linear regression step, and applying a band-pass filter (0.01–0.09 Hz) in order to reduce both noise effects and low frequency drift.

A Seed-to-Voxel parametrical analysis was performed by using four seeds of interest (3 somatomotor and 1 visual brain region, all bilaterally) that were preselected from the Harvard-Oxford atlas: precentral gyri (PreCG), postcentral gyri (PostCG), supplementary motor area (SMA), and intracalcarine cortex (ICC). The visual area was included as control. Individual correlation maps were generated extracting the mean BOLD time course from the eight preselected seeds and calculating the correlation coefficients with the BOLD time-course of each voxel throughout the whole brain. These correlations were obtained by applying the General Linear Model (GLM) and bivariate correlation analyses weighted for Hemodynamic Response Function (HRF). Only BOLD signals during successful trials were analyzed.

To examine group differences in functional connectivity during the POST compared to the PRE session, we used a 3 × 2 factorial analysis with the within-subjects factor Assessment session (PRE vs. POST) and the between-subjects factor Group (good-SMR responders, bad-SMR responders, SHAM). Furthermore, two-sample *t*-tests between pairs of groups separately for each session were performed. A whole-brain height threshold of *p* < 0.001 (uncorrected) was used to identify areas with significant functional connectivity changes, and a family-wise error (FWE)-corrected threshold of *p* < 0.01 at this height threshold was applied for all reported clusters.

## Results

### Demographic and Psychological Data

Table 1 displays demographic and psychological data of the three groups. No significant differences among groups were found on age, “years since FM was diagnosed” or depression comorbidity [χ^2^(2) = 0.049, *p* = 0.976]. One-way ANOVAs revealed slight significant differences on FIQ scores among the three groups [*F*(2, 14) = 4.156, *p* = 0.043, η_p_^2^ = 0.409]. However, Bonferroni *post hoc* analyses only showed a non-significant trend between good-SMR and bad-SMR responders on these scores (40.81 ± 5.12 and 68.37 ± 20.20, respectively, *p* = 0.062). Furthermore, one-way ANOVAs revealed significant differences among the three groups on three dimensions of the SF-36: “pain” [*F*(2, 15) = 4.116, *p* = 0.041, η_p_^2^ = 0.388], “general health perception” [*F*(2, 15) = 5.954, *p* = 0.015, η_p_^2^ = 0.478] and “change in health” [*F*(2, 15) = 7.127, *p* = 0.008, η_p_^2^ = 0.523]. Bonferroni *post hoc* analyses of these effects revealed that good-SMR responders had higher score than bad-SMR responders on the dimensions “pain” (39.75 ± 21.50 and 9.20 ± 12.60, respectively, *p* = 0.049), “general health perception” (41.25 ± 14.36 and 12.00 ± 12.55, respectively, *p* = 0.013) and “change in health” (43.75 ± 12.50 and 5.00 ± 12.18, respectively, *p* = 0.008). No significant differences among groups were observed on other self-reported questionnaires.

**TABLE 1:** Demographic data and questionnaires scores (mean ± SD) for each group, including effect sizes of the group differences (*p < 0.05; **p < 0.01).

	Good-SMR responders (*n* = 4)	Bad-SMR responders (*n* = 5)	SHAM (*n* = 8)	Effect size
**Age (years)**	54.75 ± 8.46	53 ± 9.77	56.25 ± 11.99	0.020
**FM years diagnosed (years)**	9.5 ± 6.25	12.2 ± 8.98	9.75 ± 4.46	0.038
**Depression comorbidity**	3	4	6	–
**BDI**	20 ± 7.55	35.8 ± 14.82	33.13 ± 12.4	0.198
**SF-36**				
Physical functioning	40 ± 22.73	19 ± 13.42	33.57 ± 18.42	0.202
Role limitations: physical	28.25 ± 35.95	0 ± 0	0 ± 0	–
Role limitations: emotional	29 ± 8	20 ± 27.39	12 ± 18.57	0.124
Vitality	21.25 ± 16.52	7 ± 15.65	15.71 ± 14.27	0.136
Mental health	51 ± 11.49	39.2 ± 27.77	34 ± 12.17	0.145
Social functioning	47.25 ± 11.93	15.2 ± 20.81	28.71 ± 23.77	0.292
Pain	39.75 ± 21.5^†^	9.2 ± 12.6^†^	16.29 ± 15.89	0.388*
General health perception	41.25 ± 14.36^†^	12 ± 12.55^†^	26.43 ± 11.8	0.478*
Change in health	43.75 ± 12.5^†^	5 ± 11.18^†^	17.86 ± 18.9	0.523**
**WHYMPI**				
Pain and interference				
Social support	0.5 ± 1	2.33 ± 2.43	0.42 ± 0.94	0.278
Negative Mood	3.31 ± 1.01	4.15 ± 0.8	4.03 ± 0.75	0.161
Social Interference	2.84 ± 1.5	5 ± 1.08	4.04 ± 1.29	0.311
Activity interference	4.25 ± 1.34	5.35 ± 0.95	4.66 ± 1.27	0.124
Pain Severity	3.25 ± 1.46	5.1 ± 0.78	4.22 ± 0.96	0.333
Self-Control	3.5 ± 1.22	1.6 ± 1.64	2.81 ± 1.16	0.258
Support				
Distracting	2.78 ± 1.36	2.1 ± 0.9	2.52 ± 1.76	0.033
Solicitous	3.27 ± 0.95	1.76 ± 1.28	1.95 ± 1.51	0.166
Punitive	2.89 ± 1.83	2 ± 2.01	2.46 ± 1.42	0.040
Activity interference				
Outdoor work	4.54 ± 1.56	2.67 ± 1.44	3.04 ± 1.15	0.259
Away from home	2 ± 0.73	1.08 ± 0.89	1.98 ± 1.32	0.145
Household work	0.42 ± 0.5	1.13 ± 2	1.33 ± 1.65	0.060
Social	1.59 ± 0.83	1.33 ± 1.7	1.54 ± 1.52	0.006
**MPQ**				
Sensory	10.25 ± 1.26	9.2 ± 1.64	12.88 ± 10.37	0.057
Miscellaneous	2.25 ± 0.96	3 ± 0	3.38 ± 3.2	0.043
Affective	1.25 ± 1.26	2.6 ± 0.55	2.5 ± 2.88	0.074
Evaluative	0.75 ± 0.5	1 ± 0	1.13 ± 1.25	0.031
**PASS**				
Cognitive anxiety	15.75 ± 11.56	10.92 ± 9.86	21.56 ± 13.49	0.148
Physiologic anxiety	10.15 ± 8.4	8.12 ± 8.26	16.34 ± 13.3	0.120
Escape and avoidance	13.93 ± 7.07	12.32 ± 13.54	22.29 ± 13.75	0.143
Fearful thinking	10.5 ± 11.62	11.4 ± 12.05	20.18 ± 15.86	0.115
**TSK**	27.75 ± 12.15	51.8 ± 13.72	38.13 ± 15.72	0.311
**PVAQ**	45.5 ± 8.89	51 ± 11.29	48.13 ± 12.52	0.036
**CSQ**				
Catastrophizing	9.75 ± 9.54	26.4 ± 7.37	18 ± 10.95	0.318
Increasing activity levels	14.25 ± 7.89	10.8 ± 6.61	16.38 ± 6.19	0.132
Coping self-statements	16.75 ± 8.81	12.4 ± 9.66	17.13 ± 3.27	0.099
Ignoring pain	22.25 ± 6.18	14.8 ± 8.81	23.13 ± 4.19	0.295
Reinterpreting pain	10 ± 10.86	6.2 ± 4.49	10.5 ± 7.45	0.069
Hoping	7.75 ± 3.59	9.8 ± 5.17	6.38 ± 3.5	0.135
Praying	3 ± 5.35	8.2 ± 6.8	4.38 ± 5.24	0.131
Coping self-statements	9.5 ± 1.73	8.4 ± 5.41	10.63 ± 2.88	0.077
**MOS**				
Emotional support	28.25 ± 13.57	24.4 ± 11.06	21.75 ± 6.84	0.077
Tangible support	14.5 ± 7.14	12.4 ± 3.29	11.75 ± 5.06	0.052
Positive interaction	14.5 ± 6.81	13 ± 5.2	11.75 ± 4.03	0.054
Affection	13 ± 4	11.4 ± 3.91	9.25 ± 2.55	0.207
Overall support index	70.25 ± 28.81	61.2 ± 22.71	50.75 ± 22.78	0.116
**FIQ**	40.81 ± 5.13	68.37 ± 20.2	65.16 ± 15.16	0.409*

*BDI-II, Beck Depression Inventory; SF-36, MOS 36-item Short-form Health Survey; WHYMPI, West Haven-Yale Multidimensional Pain Inventory; MPQ, McGill Pain Questionnaire; PASS, Pain Anxiety Symptoms Scale; TSK, Tampa Scale for Kinesiophobia; PVAQ, Pain Vigilance and Awareness Questionnaire; CSQ, Coping Strategies Questionnaire; MOS, MOS Social Support Survey; FIQ, Fibromyalgia Impact Questionnaire; †, good-SMR responders > bad-SMR responders (p > 0.05).*

**FIGURE 2:**
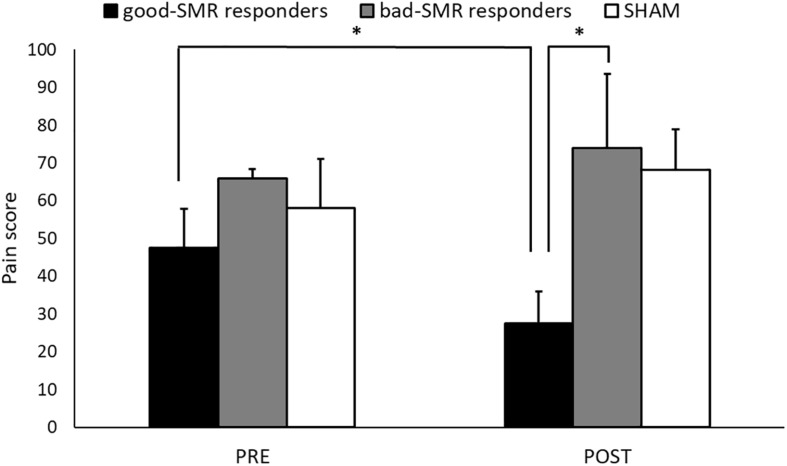
Pain ratings during the Assessment sessions (PRE and POST) for each group (^∗^indicates *p* < 0.05).

The ANOVA on pain ratings revealed a significant interaction effect of Group × Assessment Session [*F*(2, 14) = 4.103, *p* = 0.040, η*_p_*^2^ = 0.370]. The Bonferroni *post hoc* tests showed that good-SMR responders reported lower levels of pain than bad-SMR responders (27.50 ± 17.08 and 74.00 ± 19.49, respectively, *p* = 0.047) after the POST session. No significant group differences were found on pain ratings after the PRE session. Good-SMR responders also reported a significant reduction on pain ratings from the PRE to the POST session (PRE = 47.50 ± 20.62, POST = 27.50 ± 17.08; *p* = 0.042). No significant differences between the PRE and the POST sessions were observed for either bad-SMR responders (PRE = 66.00 ± 5.48, POST = 74.00 ± 19.49) or SHAM participants (PRE = 58.13 ± 36.44, POST = 68.13 ± 30.46) (Figure 2). Thus, neurofeedback training of the SMR was able to elicit a significant average pain reduction of >40% in good responders, but not in bad responders. Moreover, it was observed that all good responders (4 out of 4) reduced pain ratings, whereas neurofeedback training elicited a pain reduction in only 2 of the 5 bad responders and in 2 of the 8 participants of the SHAM group (Supplementary Table S1).

No significant differences were found due to Group or Assessment session on anxiety scores (STAI-S) or subjective ratings (pain, fatigue, and negative mood) obtained from the diary (Supplementary Table S1).

### Task Performance and EEG Neurofeedback Analyses

**FIGURE 3:**
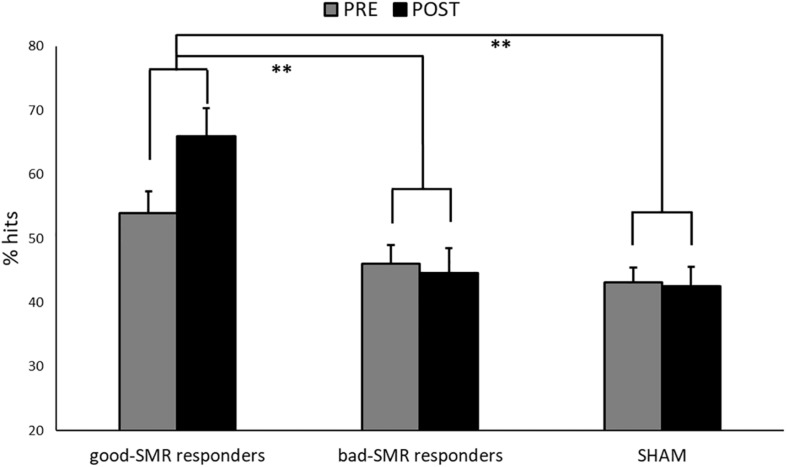
Percentage of successful trials during the Assessment sessions (PRE and POST) for each group (^∗∗^indicates *p* < 0.01).

Figure 3 displays the task performance during the PRE and the POST sessions for good-SMR responders, bad-SMR responders and the SHAM group. The good-SMR responders showed higher percentage of hits than bad-SMR responders and the SHAM group (60.25% ± 8.74, 45.30% ± 5.47, and 42.81% ± 7.40, respectively). The ANOVA on task performance revealed significant main effects of Group [*F*(2, 14) = 10.865, *p* = 0.001, η_p_^2^ = 0.608], showing significant differences between the good-SMR responders and the bad-SMR responders, as well as between the good-SMR responders and the SHAM group (Bonferroni *post hoc*: all *p*s < 0.01), but not between the bad-SMR responders and the SHAM group. No other significant effects were observed on task performance.

A similar statistical analysis of task performance was computed taking into account the Trial type (synchronization vs. desynchronization). The ANOVA results reveled significant main effects of Group [*F*(2, 14) = 10.612, *p* = 0.002, η_p_^2^ = 0.603], as well as a trend effect of Group × Assessment Session × Trial type [*F*(2, 14) = 3.300, *p* = 0.067, η_p_^2^ = 0.320]. The Bonferroni *post hoc* test yielded significant differences in bad-SMR responders and the SHAM group between trial types during the POST session (all *p*s < 0.01). Thus, percentage of hits during synchronization were higher than during desynchronization in bad-SMR responders (62.80% ± 10.92 and 26.00% ± 15.75, respectively) and the SHAM group (53.75% ± 7.74 and 31.25% ± 11.16, respectively) during the POST session. In contrast, good-SMR responders showed no significant differences in percentage of hits between synchronization and desynchronization during the POST session (67.00% ± 13.11 and 62.50% ± 15.61, respectively, *p* = 0.614). During the PRE session, only the SHAM group showed significant differences in percentage of hits between synchronization and desynchronization (53.75% ± 9.35 and 32.25% ± 14.04, respectively, *p* = 0.003) (Supplementary Figure S1).

The duration of successful trials during synchronization and desynchronization trials in the assessment sessions are shown for the three groups in Supplementary Figure S2. The ANOVA revealed a significant effect of Group × Trial type [*F*(2, 14) = 6.064, *p* = 0.013, η_p_^2^ = 0.464]. Bonferroni *post hoc* test showed that desynchronization trials lasted significantly longer than the synchronization trials in bad-SMR responders (4.15 s ± 0.81 and 3.15 s ± 0.79, respectively) and the SHAM group (4.03 s ± 0.77 and 3.33 s ± 0.50, respectively) (all *p*s < 0.001). In contrast, no significant differences were found on duration of successful trials between desynchronization and synchronization in good-SMR responders (3.83 s ± 0.66 and 3.82 s ± 0.62, respectively) (*p* = 0.958). No other significant differences due to group, Assessment session or Trial task were found.

**FIGURE 4:**
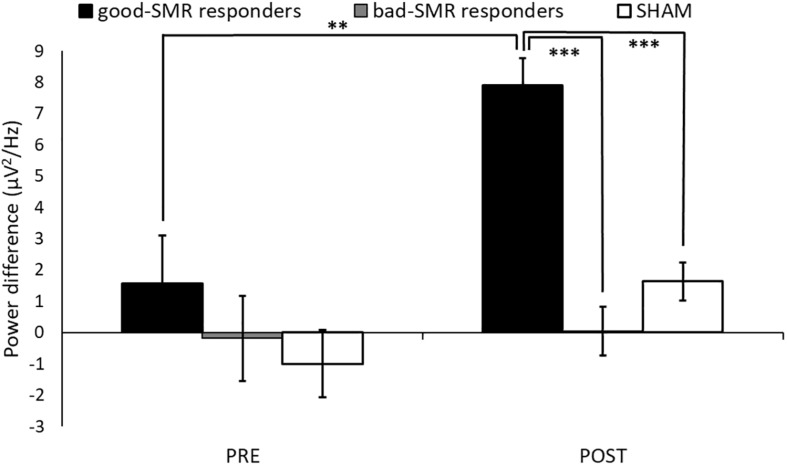
SMR modulation score (power difference between synchronization and desynchronization) over C3, CP1 and CP5 electrodes during the Assessment sessions (^∗∗^ and ^∗∗∗^ indicate *p* < 0.01 and *p* < 0.001, respectively).

Figure 4 displays changes of SMR power modulation (difference between synchronization and desynchronization trials at electrodes C3, CP1, and CP5 within 12–15 Hz) during the Assessment sessions. The ANOVA revealed significant main effects of Group [*F*(2, 14) = 11.129, *p* = 0.001, η_p_^2^ = 0.614], as well as a trend effect of Group × Assessment Session [*F*(2, 14) = 3.225, *p* = 0.070, η_p_^2^ = 0.315]. The Bonferroni *post hoc* tests showed significant differences between good-SMR and bad-SMR responders, as well as between good-SMR responders and the SHAM group during the POST session (all *p*s < 0.001). SMR modulation score was higher in good-SMR responders (7.91 μV^2^/Hz ± 2.20) than in bad-SMR responders (0.04 μV^2^/Hz ± 1.06) and SHAM group (1.63 μV^2^/Hz ± 1.81). No significant group differences were found during the PRE session. Furthermore, only good-SMR responders displayed a significant enhancement of the SMR modulation score between the PRE (1.57 μV^2^/Hz ± 1.41) and the POST sessions (7.91 μV^2^/Hz ± 2.20) (*p* = 0.003).

Finally, correlational analyses revealed that percentage of successful trials during the POST session was positively correlated with the “pain” (*r* = 0.688, *p* = 0.003) and “change in health” (*r* = 0.715, *p* = 0.002) dimensions of the SF-36 questionnaire. Furthermore, SMR modulation was negatively correlated with FIQ scores (*r* = -0.552, *p* = 0.033). No correlations were found between performance scores, SMR modulation and questionnaire scores during the PRE session.

### Functional MRI Data

The hypothesis that participants with a successful SMR neurofeedback training would show an increased somatomotor functional connectivity was explored using a seed-to-voxel analysis. No significant group differences were found on functional connectivity from PRE to POST sessions. Nevertheless, significant group differences on the functional connectivity of the somatomotor seed regions with a variety of cortical regions were observed during both the POST and the PRE sessions, separately. Table 2 shows the T-maxima of the significant clusters, as well as MNI coordinates, *P*-values (FWE corrected) and the size of each cluster in contiguous voxels for PRE and POST sessions.

**TABLE 2:** Seed-to-voxel results of the two-sample *t*-tests comparing pairs of groups during the PRE and POST sessions.

Seed	Contrast pair	Cluster [x,y,z]	k	Cluster p-FWE	Peak p-unc	Result region
**PRE**						
PreCG L	Bad-SMR responders > SHAM	−44 −44 −04	434	0.000000	0.000003	Temporooccipital middle temporal gyrus L Angular gyrus L Inferior lateral occipital cortex L
		−64 −20 +18	234	0.000031	0.000000	Central opercular cortex L Planum temporale L Anterior supramarginal gyrus L Parietal operculum L Anterior superior temporal gyrus L
PostCG L	Bad-SMR responders > SHAM	−32 −68 +10	1122	0.000000	0.000003	Superior lateral occipital cortex L Inferior lateral occipital cortex L Temporooccipital middle temporal gyrus L Angular gyrus L
ICC R	Bad-SMR responders > SHAM	−38 −58 +48	393	0.000000	0.000001	Superior lateral occipital cortex L Angular gyrus L Superior parietal lobule L
		−14 +38 +48	317	0.000005	0.000007	Superior frontal gyrus L Middle frontal gyrus L
**POST**						
PreCG L	Bad-SMR responders > good-SMR responders	−06 −42 +44	301	0.000002	0.000001	Precuneous Posterior cingulate gyrus L
PostCG R	Good-SMR responders > SHAM	−46 −32 +58	458	0.000002	0.000020	Postcentral gyrus L Anterior supramarginal gyrus L
	Bad-SMR responders > good-SMR responders	+42 −62 +44	399	0.000000	0.000005	Angular gyrus R Superior lateral occipital cortex R
PostCG L	Good-SMR responders > SHAM	+28 −24 +56	1426	0.000000	0.000000	Postcentral gyrus R Precentral gyrus R Anterior supramarginal gyrus R Superior parietal lobule R
		+38 −38 −14	588	0.000000	0.000003	Temporal occipital fusiform cortex R Lingual gyrus R Posterior temporal fusiform cortex R Temporooccipital inferior temporal gyrus R Posterior parahippocampal gyrus R Posterior inferior temporal gyrus R
		+52 −32 +36	198	0.000993	0.000042	Posterior supramarginal gyrus R
	Good-SMR responders > bad-SMR responders	+50 −14 +50	1175	0.000000	0.000001	Postcentral gyrus R Precentral gyrus R Superior parietal lobule R
	Bad-SMR responders > good-SMR responders	−30 −10 +34	274	0.000003	0.000008	Insular cortex L
	Bad-SMR responders > SHAM	−52 −60 +24	472	0.000000	0.000001	Superior lateral occipital cortex L Angular gyrus L

*A whole-brain height threshold of p < 0.001 (uncorrected) was used to identify areas with significant functional connectivity changes, and a family-wise error (FWE)-corrected threshold of p < 0.01 at this height threshold was applied for all reported clusters (PreCG, precentral gyrus; PostCG, postcentral gyrus; ICC, intracalcarine cortex; L, left; R, right).*

During the PRE session, bad-SMR responders (compared to SHAM group) exhibited increased functional connectivity between the left PreCG seed and the left temporooccipital middle temporal gyrus [*t*(11) = 7.95, *p* < 0.001] and the left central opercular cortex [*t*(11) = 9.93, *p* < 0.001], as well as between the left PostCG seed and the superior lateral occipital cortex [*t*(11) = 8.20, *p* < 0.001] and the left temporooccipital middle temporal gyrus [*t*(11) = 6.66, *p* < 0.001], and between the right ICC seed and the superior lateral occipital cortex [*t*(11) = 9.19, *p* < 0.001] and the left superior frontal gyrus [*t*(11) = 7.36, *p* < 0.001]. No significant differences on seed-to-voxel connectivity were found between good-SMR and bad-SMR responders, or between good SMR responders and SHAM in the PRE session.

**FIGURE 5:**
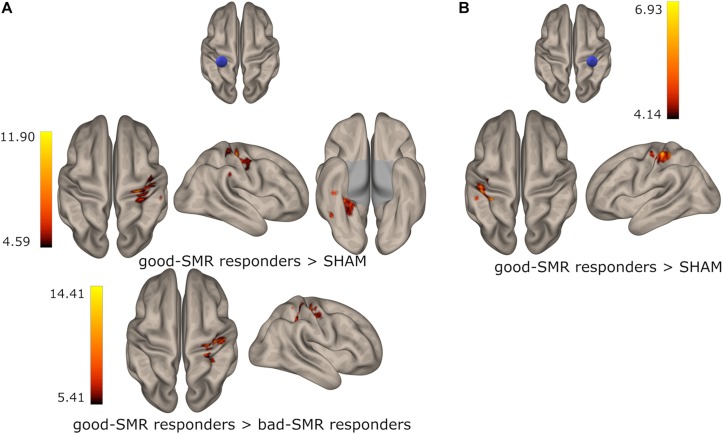
Group differences on functional connectivity of the left **(A)** and the right **(B)** postcentral gyrus with somatomotor areas in the POST session. Color indicates the connectivity strength in good-SMR responders when compared to bad-SMR responders and the SHAM group.

During the POST session, good-SMR responders (compared to SHAM) exhibited increased functional connectivity between the right PostCG seed ant the left PostCG [*t*(10) = 6.93, *p* < 0.001] and between the left PostCG seed and the right PostCG [*t*(10) = 11.90, *p* < 0.001] and the right temporal occipital fusiform cortex [*t*(10) = 8.59, *p* < 0.001]. Good-SMR responders (compared to bad-SMR responders) also demonstrated enhanced connectivity between the left PostCG seed and the right PostCG [*t*(7) = 14.41, *p* < 0.001]. Furthermore, bad-SMR responders (compared to good-SMR responders) presented increased functional connectivity between left PreCG seed and the precuneous [*t*(7) = 14.16, *p* < 0.001] and between right PostCG seed and the right superior lateral occipital cortex [*t*(7) = 11.09, *p* < 0.001]. Bad-SMR responders (compared to SHAM) also demonstrated enhanced connectivity between the left PostCG seed and the left superior lateral occipital cortex [*t*(11) = 8.80, *p* < 0.001]. Thus, it appears that good-SMR responders had improved functional connectivity among somatomotor areas during the POST session; whereas bad-SMR responders showed increased functional connectivity with visual areas. Figure 5 displays those brain locations, where the functional connectivity of the left and right PostCG seed was higher in good-SMR than in bad-SMR responders and SHAM.

## Discussion

The aim of the present study was to examine changes in SMR power and functional connectivity of the somatosensory and motor cortices during a neurofeedback training based on synchronization and desynchronization of the SMR power over motor and somatosensory areas in patients with fibromyalgia (FM). In addition, changes in fMRI connectivity of somatosensory and motor cortices elicited when performing the neurofeedback training were also analyzed. Participants were randomly assigned to a SMR training group (real feedback) or to a SHAM group (non-contingent feedback). The analyses of the task performance during the six sessions revealed that only some participants of the SMR group were able to achieve a success rate above 50% (chance level). Thus, SMR group participants were further subdivided into good (good-SMR responders, those participants who performed the task above the chance level), and bad responders (bad-SMR responders, those participants who performed the task at the chance level). Good responders displayed significant enhancements of power modulation (the difference between SMR synchronization and desynchronization) at electrodes over somatomotor cortices, as well as increased functional connectivity between motor and somatosensory related areas during the last session as compared to the first session of the neurofeedback training. No changes on brain activity or connectivity were observed in bad responders or in the SHAM group. In addition, good responders significantly reduced pain ratings compared to both bad responders and the SHAM group.

Taking together all participants who received SMR neurofeedback training, it was observed that their average percentage of hits during the task was similar to the SHAM group and close to the random probability level. This finding contrasts with previous studies showing that healthy participants can learn to modulate SMR in one session and achieve a successful performance in the neurofeedback task of around 75% (Popescu et al., 2007; Blankertz et al., 2008, 2010). In the present study, we decided to examine the effects of a six-session neurofeedback training program (3 sessions per week for 2 weeks) that was already tested in healthy participants with a successful performance of above 75% (Terrasa et al., 2019). The lack of information about the success rate in previous studies with fibromyalgia patients (Mueller et al., 2001; Kravitz et al., 2006; Kayıran et al., 2010; Nelson et al., 2010; Caro and Winter, 2011) makes difficult the comparison, but it is worthy to highlight that about 20% of healthy individuals cannot modulate their cerebral activity (Allison and Neuper, 2010). In our study, around half of FM participants were not able to perform successfully the neurofeedback task, and we decided to subdivide the participants who received the SMR training in good and bad responders to further explore the differences in brain activity and functional connectivity. Even good responders achieved an average success rate of above 60% in the neurofeedback training task, below the performance previously observed in healthy subjects. Thus, it seems that the presence of chronic pain could affect the behavioral performance in neurofeedback tasks. In this sense, we also observed that good and bad responders to the SMR neurofeedback training displayed significant differences in several clinical characteristics of pain symptoms before the training program. Thus, for instance, good-SMR responders had significant lower scores in pain impact (FIQ and SF-36 dimension), together with better health perception and health change (SF-36 dimensions) than bad-SMR responders. By contrast, participants in the SHAM group yielded better scores on health status and pain impact than bad responders, but worse than good responders. In addition, significant correlations were observed between successful performance in the neurofeedback task and pain impact and perceived health status, indicating that only those FM participants with less symptom severity were able to perform successfully the neurofeedback training. Other pain-related symptoms such as depression, anxiety or kinesiophobia were not relevant for task performance. These data suggest that the poor performance of chronic pain patients could be related to the direct impact of chronic pain on their health rather than to other pain comorbidities. Future research should further clarify the role of chronic pain in the performance of the neurofeedback task and explore whether there are some patients who could benefit more than others from neurofeedback training.

The best performance of good responders was also reflected in the ability to synchronize and desynchronize SMR with the same success and speed. On the other hand, bad responders and participants in the SHAM group were worse and slower to desynchronize than to synchronize the SMR. Moreover, there was no modulation of SMR power (difference between EEG power in synchronization and desynchronization trials) neither in bad responders nor in the SHAM group. Thus, it appears that the optimal task performance was accompanied by a better power modulation of both synchronization and desynchronization of the SMR over motor and somatosensory related electrodes after the neurofeedback training. Our findings are in agreement with previous data showing, for instance, that FM patients were able to modulate delta, theta and alpha EEG power (Mueller et al., 2001), or the theta/SMR ratio after the neurofeedback training (Kayıran et al., 2010). Considering that most neurofeedback studies have reported significant but unspecific changes in brain activity over the cortex, the present study provides further evidence that only some FM participants were able to self-regulate their brain activity over somatomotor cortices and that these modulatory changes were followed by a significant reduction in pain.

Previous studies have suggested that neurofeedback training may increase resting-state functional connectivity in several pain areas such as ACC (Ros et al., 2013), insula (Kluetsch et al., 2014) or the amygdala (Nicholson et al., 2016). In addition, significant enhancements of resting-state functional connectivity of somatosensory and motor cortices have been demonstrated in patients with stroke (Várkuti et al., 2013; Young et al., 2014; Mohanty et al., 2018) and healthy participants (Terrasa et al., 2019). In the present study, significant changes in functional brain connectivity of motor and somatosensory areas were observed when performing the neurofeedback task in good responders to the neurofeedback training of the SMR, but not in bad responders or participants in the SHAM group. Indeed, good responders showed higher functional connectivity of the bilateral PostCG with other somatosensory and motor areas than bad-SMR responders and the SHAM group. Therefore, it appears that a successful neurofeedback training based on the modulation of the SMR may lead to a greater interconnectivity between somatosensory and other somatomotor areas. In contrast, bad responders displayed increased functional connectivity between somatomotor areas and several brain areas during both before and after the neurofeedback training. Most of these areas (precuneous, angular gyrus and superior lateral occipital cortex) are involved in visuospatial processing and object recognition (Grill-Spector et al., 2001; Cavanna and Trimble, 2006; Seghier, 2013), suggesting that bad responders were trying to use some visual strategy to solve the neurofeedback task. Interestingly, functional connectivities of somatomotor areas and the insula, as well as visual processing and pain-related areas, were also increased in bad responders at the beginning and the end of the neurofeedback training. It is well known that the insula is involved in sensory and affective dimensions of pain processing and its functional connectivity seems to be impaired in chronic pain leading to a disruption of modulatory circuits involved in pain (Lu et al., 2016). Thus, our findings of an enhanced functional connectivity of these pain-related brain areas in bad responders suggest that patients could be more focused on pain perception, rather than on the neurofeedback task.

Together with changes in activity and functional connectivity within motor and somatosensory brain areas, neurofeedback training of the SMR was able to elicit a significant average pain reduction of >40% (2 cm on the VAS) in good responders, but not in bad responders. In addition, it was observed that all good responders (4 out of 4) reduced pain ratings, whereas neurofeedback training elicited a pain reduction in only 2 of the 5 bad responders. Although the sample of the present study was small, our findings are in agreement with previous studies showing that neurofeedback training of SMR can produce pain reduction in chronic pain patients (Mueller et al., 2001; Kayıran et al., 2010; Nelson et al., 2010; Caro and Winter, 2011). Furthermore, our neurofeedback training protocol consisted of six sessions, while other studies with relevant clinical effects have used at least 10 (Kayıran et al., 2010), or even more than 30 sessions (Caro and Winter, 2011). Indeed, there is significant variability in terms of study design and intervention procedures (duration and number of treatment sessions) with respect to neurofeedback intervention in patients with fibromyalgia (Santoro and Cronan, 2014). In the present study, we decided to examine the effects of a six-session neurofeedback training program (3 sessions per week for 2 weeks) that was already tested in healthy participants (Terrasa et al., 2019). Although patients with chronic pain achieved poorer performance than healthy controls in this program, our findings seem to indicate that 4–6 training sessions may be enough to produce positive clinical results. Thus, this short program could be used as a marker to examine the long-term suitability of neurofeedback training to reduce pain in patients with chronic pain.

Nevertheless, the design of the present study has some shortcomings and its findings should be taken with caution. First and most important, the sample size was small and this makes the findings only preliminary, especially in the fMRI analyses. Second, the fact that all participants took regular medication during neurofeedback training could have biased the results, so their possible effects on the brain changes observed in this study should be further explored. Third, all subjects were women and, therefore, further studies should include male participants with FM to assess the possible influence of gender on the effects of neurofeedback training. Fourth, this was not a double-blind study and, therefore, our findings could be affected by factors that were not related to the neurofeedback intervention. And finally, our psychological assessment was designed to characterize patients and ensure that the groups were comparable in those measures before the training. Further analyzing the effects of neurofeedback training on self-report questionnaires (including depression) is of great interest and should be examined in subsequent studies.

In summary, the present study revealed that neurofeedback training based on the synchronization and the desynchronization of the SMR led to an augmented functional connectivity between areas associated with the somatosensory and motor activity, as well as to an enhancement of power modulation in fibromyalgia patients. Nevertheless, this result was only obtained in those participants with less impact of the fibromyalgia symptoms. Moreover, these changes in EEG power and functional brain connectivity were mirrored by a reduction in pain. In this sense, our research provide evidence that neurofeedback training is a promising tool for a better understanding of brain mechanisms involved in pain chronification.

## Data Availability Statement

The datasets generated for this study are available on request to the corresponding author.

## Ethics Statement

The studies involving human participants were reviewed and approved by the Ethics Committee of the Balearic Islands (Spain). The patients/participants provided their written informed consent to participate in this study.

## Author Contributions

JT, AB-L, PM, and MM contributed significantly to the design of the study. JT collected the data. JT and AB-L performed the data analyses. JT and MM wrote most of the manuscript. AB-L and PM critically revised the manuscript.

## Conflict of Interest

The authors declare that the research was conducted in the absence of any commercial or financial relationships that could be construed as a potential conflict of interest.

## Footnotes

1 http://www.fil.ion.ucl.ac.uk/spm/
