# Supplementary material for: Self-Regulation of SMR Power Led to an Enhancement of Functional Connectivity of Somatomotor Cortices in Fibromyalgia Patients
Source: Front Neurosci. 2020 Mar 19;14:236. doi: 10.3389/fnins.2020.00236 (PMC7103632; doi:10.3389/fnins.2020.00236)
Supplement: Supplementary file 1 [file Table_1.DOCX]

Supplementary Material

# Supplementary Figures and Tables

| **Subject code** | **Group** | **PRE** (0-100) | **POST** (0-100) | **Percentage of pain reduction** |
| --- | --- | --- | --- | --- |
| 105 | good-SMR responders | 50 | 30 | -40% |
| 113 | good-SMR responders | 50 | 20 | -60% |
| 116 | good-SMR responders | 20 | 10 | -50% |
| 121 | good-SMR responders | 70 | 50 | -29% |
| 101 | bad-SMR responders | 60 | 50 | -17% |
| 107 | bad-SMR responders | 70 | 100 | 43% |
| 111 | bad-SMR responders | 60 | 80 | 33% |
| 115 | bad-SMR responders | 70 | 60 | -14% |
| 117 | bad-SMR responders | 70 | 80 | 14% |
| 100 | SHAM | 95 | 85 | -11% |
| 103 | SHAM | 0 | 0 | 0% |
| 108 | SHAM | 10 | 60 | 500% |
| 109 | SHAM | 70 | 80 | 14% |
| 112 | SHAM | 50 | 80 | 60% |
| 119 | SHAM | 70 | 80 | 14% |
| 120 | SHAM | 70 | 60 | -14% |
| 122 | SHAM | 100 | 100 | 0% |

**Supplementary Table 1.** Individual pain ratings (0-100 numeric scale) after the PRE and POST assessment sessions. The percentage of pain reduction was calculated as the percentage of the POST/PRE quotient.


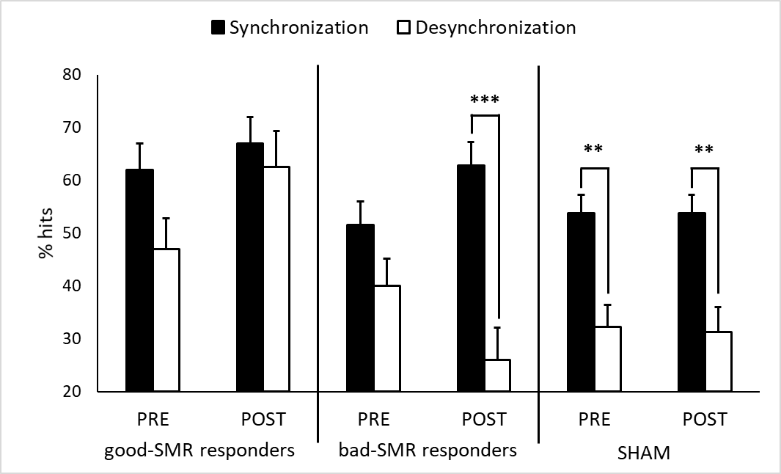


**Supplementary Figure 1.** Percentage of successful trials separated by trial type (synchronization or desynchronization) during the Assessment sessions (PRE and POST) for each group (** and *** indicate p< .01 and p< .001, respectively).


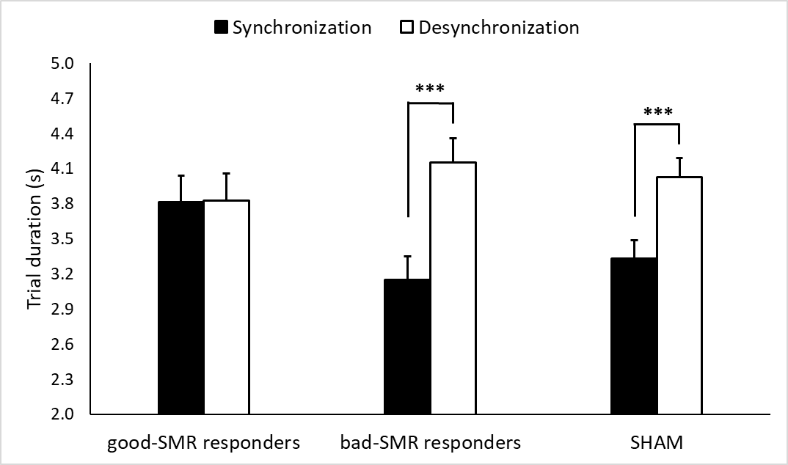


**Supplementary Figure 2.** Duration (seconds) of successful synchronization and desynchronization trials during the Assessment sessions (PRE and POST) for each group (*** indicates p< .001).
